# Surgery

**Published:** 1899-12

**Authors:** W. M. Barclay


### SURGERY.

In the *Bristol Medico-Chirurgical Journal* for 1895,[4] I gave a summary of some remarkable results of **extensive operations for cancer of the breast,** as advocated and practised especially by Watson Cheyne, Halsted, and Meyer. As two of these surgeons have brought their statistics up to date, and given us the results of their further experience; and as some new pathological evidence has been adduced, it may be useful to review the subject afresh.

The pathological evidence is mainly embodied in a very important and exhaustive paper on the dissemination of cancer of the breast, by Mr. Stiles,[5] which is an eloquent substantiation of the radical operations they have urged on the profession. He criticises the opinion that so largely prevailed in the discussion which followed Mr. Marmaduke Sheild's paper read at the Royal Medical and Chirurgical Society last year,[6] by pointing out that the ultimate prognosis for patients free from recurrence three years after the old operation cannot be as good as when the same immunity has followed a modern extensive operation, since in the latter case the risk is largely limited to internal recurrence.

To begin with Mr. Cheyne's statistics. He gives the present condition, as far as he can ascertain it, of the 61 cases reported[7]

1 *J. Nerv. & Ment. Dis.*, 1897, xxiv. 67.

2 *Psychiat. en Neurol. Bl.*, 1898, ii. 118; abstract in *Neurol. Centralbl.*, 1899, xviii. 855.

3 *Riv. quindicin. di psicol.* [etc.], 1898, ii. 213; abstract in *J. Nerv. & Ment. Dis.*, 1899, xxvi. 196.

4 xiii. 29.

5 *Brit. M. J.*, 1899, i. 1452.

6 *Ibid.*, 1898, i. 300, 557.

7 *Lancet*, 1896, i. 397 *et seq.*

by him more than three years ago, which then covered a period of six years. In 21 of these three years or more had elapsed since operation, and 12 of them (57 per cent.) had remained free from recurrence, 1 having died of an independent illness three years and five months after operation. Of these 11 patients 9 are reported as being still well, 1 was alive and well a year ago (seven years after operation), but has since been lost sight of, and 1 has had a recurrence of the disease. If a patient operated upon more than nine years ago (and who was not included among the 21 cases), and who was recently seen with no signs of recurrence, be added to the number, the percentage of successes remains practically the same as before. Of the 40 cases under the three-year limit, 27 had shown no sign of recurrence when the last paper was written, and 18 of these are still alive and well, while 1 died without sign of recurrence. Mr. Cheyne points to these figures as a corroboration of Volkmann's well-known dictum, since of 11 patients reported well three to six years after operation, only 1 has recurred in the succeeding three years, while of 20 who had survived the operation a year at his last writing 17 still remain well, thus showing, as he believes, the great chance of freedom from recurrence which patients obtain who pass one year without a return of the disease after a radical operation. To his original 61 cases, Mr. Cheyne adds 38 fresh ones in which one to three years have elapsed since operation, and 26 of these remain well.

Dr. Halsted[1] gives the following statistics of the cases operated upon at the Johns Hopkins Hospital by his method up to April, 1898. They are 133 in number. There have been 13 (9 per cent.) "local" and 22 (16 per cent.) "regionary" recurrences. Of 76 cases operated upon three or more years ago, 31 (40 per cent.) are living and well; 10 died more than three years after operation, and 1 as late as five and a half years after. 35 cases (46 per cent.) died within three years of the operation.

In his recent paper Mr. Cheyne calls special attention to certain points, which may be taken categorically, and discussed in the light of clinical and pathological knowledge.

*Exploration in Doubtful Cases.*—He strongly deprecates an incision into the tumour in such a case, and asserts again that the cancer cells may escape and infect the wound afterwards made for removal of the breast. He says he has more than once seen a diffuse cancerous infection, especially when glands have burst during removal, and in spite of the greatest care in clearing out all the escaped material. Hence he recommends that the suspected swelling should be "excised along with an area of apparently healthy tissue around," and only cut into away from the site of operation; that it should not be touched by the surgeon's fingers; and that all instruments employed should be

[1] *Ann. Surg.*, 1898, xxviii. 557.

discarded. To guard against the risk of contamination from lymphatic channels cut across, Mr. Cheyne, after stopping the bleeding, stuffs the wound with a small sponge and stitches it closely up, and then disinfects his hands and the patient's skin as thoroughly as in the first instance, before proceeding to remove the breast. Dr. Halsted recommends the same scrupulous avoidance of an incision into a suspected tumour.

*Skin Incisions.*—Mr. Cheyne believes that in the free removal of the skin—quite independently of the question of closure of the wound afterwards—lies one of the chief reasons why he has so seldom had local recurrences; and he gives a useful series of diagrams representing the incisions he employs in different positions of the growth—the skin over the breast always being removed, and when the growth is excentral the incision being planned to keep wide of it. Then the skin is undermined all round (leaving just enough fat with it to ensure its vitality) as far as the clavicle above, beyond the middle of the sternum internally, down to the origin of the abdominal muscles below, and out to the edge of the latissimus dorsi—and not till these limits are reached are the muscular fibres exposed. By this undermining (sometimes still further extended for the purpose) it appears that the edges of even these large wounds can be brought together as a rule, but when this cannot be done the unclosed part is skin-grafted at once. Dr. Halsted[1] says: "We remove rather more skin than we did originally, and in all cases we graft the wound immediately." As bearing on the importance of such free removal of skin and fat, I may quote Mr. Stiles's observations.[2] He describes the suspensory ligaments of Cooper as being tooth-like processes of breast-tissue ending in fibrinous prolongations into which the parenchyma is prolonged—these processes branching and joining to form a reticular network, while from the circumference of the gland similar processes radiate between the lobules of the circummammary fat: and in both cases lymphatic vessels are carried with them which are directly continuous with those in the skin and subcutaneous tissue of the whole mammary area. Mr. Stiles has often demonstrated the presence of disseminated foci of disease upon the exposed surface of the parts removed, and he has found cancerous lymphatic emboli towards the inner margin of the breast when the primary tumour was situated in the outer hemisphere, and *vice versâ*. At other times cancerous foci are found beneath the skin, or in the corium, which can sometimes be shown to be at the end of a paravascular lymphatic in a ligament of Cooper. In this connection Mr. Stiles states that it is not always easy to say whether a cancerous embolus is in the paravascular lymphatic or in a small vein itself, and he quotes Goldmann as having demonstrated that blood-vessels, especially small veins, are more frequently and more extensively invaded (from without) than has been hitherto supposed. More rarely

1 *Loc. cit.* 2 *Loc. cit.*

the arteries are invaded, and he suggests that possibly the sudden formation and extensive distribution of lenticular dissemination in the skin may sometimes be due to arterial embolism. Goldmann also declares that the lactiferous ducts may be invaded and give rise to a condition indistinguishable from duct cancer.

*Pectoral Muscle.*—There is no longer any difference of opinion as to the necessity of removing the pectoral fasciæ, but the controversy with regard to the treatment of the muscles themselves is still unsettled. Rotter[1] examined the pectoral muscle after removal of the breast in 40 cases, and found that the branches which perforate the pectoralis major into the retromammary fat, and into the breast itself (branches of the internal mammary, superior thoracic, and long thoracic arteries) were accompanied by efferent lymphatic vessels from the breast. He also found that one or two small glands were constantly present alongside the trunk of the superior thoracic artery, while in half the specimens examined others were situated in the angles of bifurcation of its branches, not far from the perforating branches; and in a few instances one was discovered in the substance of the pectoralis in relation to one of the same arteries. In rather more than a third of the carcinomatous breasts examined he found small cancerous nodules upon the posterior surface and in the substance of the pectoralis major, adjacent to the superior thoracic artery. It is worthy of notice that out of the 15 preparations in which the retropectoral glands were diseased, only 5 showed direct invasion of the muscle of the tumour—a layer of retromammary fat separating the two in the other 10 cases; while, on the other hand, of the 18 cases in which there was no evidence of retropectoral infection the tumour in the breast was adherent to the muscle in 5 cases. Rotter draws the following conclusions from these observations :—(1) That when the pectoral muscle is directly invaded by the tumour it is not infected throughout but through the main paravascular lymphatics. (2) That at an early stage, before any adhesion has taken place between the breast and the underlying pectoral fascia, cancer cells may be conveyed through the muscle to the glands in the retropectoral fascia, and this in about one-third of the cases. (3) That these efferent lymphatics only pass through the sternal portion of the muscle, and that the clavicular portion need not be removed unless large cancerous infraclavicular glands have become adherent to it. Dr. Halsted removes both pectoral muscles before exposing the subclavian vein; whence the axilla is stripped of its contents from within outward and from above downward. Cheyne removes the lower part, "say half of the muscle," in all cases—that is to say, all the muscle which lies behind the cancer from a little above the cancer down to its lower edge; and he only takes away the whole of the muscle, or even the whole of its pectoral origin,

[1] Quoted by Stiles *loc. cit.*

when there is evident disease of the muscle itself. He asserts that when the lower half of the pectoralis major has been taken away it is quite easy to completely expose the axilla, and that the removal of the pectoralis minor is quite unnecessary for that purpose. The fasciæ covering both sides of the two pectoral muscles should be removed as a matter of routine. In clearing out the axilla the fat and glands are dissected off as much as possible *en masse*, with the superficial fibres of the serratus magnus muscle, and the fascia over the latissimus dorsi, up to the level of the vessels; then the fat is dissected off the front of the vessels and nerves, opening and stripping the sheath; and finally the space behind them is cleared. Stiles recommends removing the branches of the axillary vein which come to it from the chest, on account of their lymphatic accompaniments.

*The Supraclavicular Glands.*—Two questions arise here: whether and when it is necessary to clear out the posterior triangle of the neck; and how far supraclavicular gland infection is a contra-indication to operation. Cheyne points out that the lymphatic course which passes upwards behind the axillary vessels to the glands in the posterior triangle of the neck is decidedly the less frequent one for cancerous infection: hence his rule is—assuming that there is no palpable enlargement—not to clear out the posterior triangle unless he finds enlarged glands in the fat running up behind the vessels. He adds that, as a matter of experience, he has hardly ever seen a recurrence in the supraclavicular glands. On the other hand, Dr. Halsted's rule is to "operate on the neck in every case." In 67 such operations cancer was found microscopically in 23 cases (34 per cent.), while in 14 more its absence had not yet been exhaustively ascertained. Fourteen of these operations were performed for palpable glands after the original breast removal, and 4 of the patients are living and free from recurrence: 2 more than four years, and the other 2 three and a half and three years after the primary operation. Dr. Bloodgood[1] is reported to have done as many as three operations for glandular involvement in the neck in two instances, and "apparently" saved both patients.

Dr. Cushing[1] cleaned out the anterior mediastinum on one side for recurrent cancer in three instances; but the results are not stated. Dr. Halsted's comment, however, is that in the near future he expects the anterior mediastinum to be cleared out at some of the primary operations. He hopes that we shall not abandon as hopeless all cases in which there is supraclavicular gland infection. Mr. Cheyne is much more pessimistic. He doubts the expediency of operation when there is marked enlargement of cervical glands in front of or beneath the sterno-mastoid; but if there is enlargement of gland "in the posterior part of the posterior triangle" he does not think it necessarily a contra-indication, provided the patient is in a state to stand a prolonged operation. Time and further experience can alone

1 Quoted by Halsted, *loc. cit.*

determine the best routine practice in this respect. Mr. Cheyne's last word may fittingly close this review of the subject: "I may add my firm conviction that *the patient's chance lies in the first operation.*" Secondary operations, as we all sorrowfully have to admit, are seldom successful.

To the pathological side of breast cancers, Dr. Halsted[1] contributes an interesting account of certain **adeno-carcinomata,** of which he has met 5 or 6 in less than 150 cases of cancers of the breast. These adeno-carcinomata (which are minutely described and figured) resembled on section the carcinomata, without any villous or papillomatous tendency: in some cases fine, worm-like cylinders of epithelium could be expressed from the cut surface; all infiltrated the surrounding tissues just as the other carcinomata do. Microscopically the power of the epithelium to make ring-like combinations was very conspicuous. Clinically they seem to be characterised by a tendency to exuberance and fungation, and sometimes to distinct pedunculation; and when fungating, a serous fluid can sometimes be squeezed from the tumour: the glands in the axilla, although enlarged, seem never to show any evidence microscopically of malignant infection, but always of endothelial proliferation; and they seem to exhibit a relatively low malignancy.

In some a change was evident from adeno-carcinoma to the purer carcinoma; and one case is reported of a true scirrhus starting in the wall of a cyst, which surrounded a little field of intracanalicular papillomatous adeno-carcinoma, which in certain parts resembled the variety of adeno-carcinoma just described, but more closely recalls the duct-cancers.

There would seem, therefore, to be several transitional varieties between the pure carcinoma and the pure adeno-carcinoma. Clinically it will be well to bear in mind this occasional and less malignant variety of breast-cancer.

W. M. BARCLAY.

